# MRI-Guided Electrophysiology Intervention*

**DOI:** 10.5041/RMMJ.10015

**Published:** 2010-10-31

**Authors:** Henry R. Halperin, Aravindan Kolandaivelu

**Affiliations:** Johns Hopkins University, School of Medicine, Division of Cardiology, Baltimore, MD 21205, USA

**Keywords:** magnetic resonance imaging, ablation, electrophysiology, arrhythmia

## Abstract

Catheter ablation is a first-line treatment for many cardiac arrhythmias and is generally performed under X-ray fluoroscopy guidance. However, current techniques for ablating complex arrhythmias such as atrial fibrillation and ventricular tachycardia are associated with sub-optimal success rates and prolonged radiation exposure. Pre-procedure 3-D magnetic resonance imaging (MRI) has improved understanding of the anatomic basis of complex arrhythmias and is being used for planning and guidance of ablation procedures. A particular strength of MRI compared to other imaging modalities is the ability to visualize ablation lesions. Post-procedure MRI is now being applied to assess ablation lesion location and permanence with the goal of identifying factors leading to procedure success and failure. In the future, intra-procedure real-time MRI, together with the ability to image complex 3-D arrhythmogenic anatomy and target additional ablation to regions of incomplete lesion formation, may allow for more successful treatment of even complex arrhythmias without exposure to ionizing radiation. Development of clinical grade MRI-compatible electrophysiology devices is required to transition intra-procedure MRI from preclinical studies to more routine use in patients.

## INTRODUCTION

Radiofrequency (RF) catheter ablation has advanced over the last 25 years from an experimental procedure to the first-line treatment for a number of cardiac arrhythmias including atrioventricular re-entrant tachycardia, accessory pathway-associated tachycardias, and typical atrial flutter.^1^ These procedures are typically guided by positioning electrode catheters using X-ray fluoroscopy and using these catheters to observe the propagation of electrical activity through the heart. Successful targeting of ablation primarily to the anatomic arrhythmia substrate, as opposed to mapping and targeting ablation based on electrogram characteristics, began with recognition that common atrial flutter passes through a narrow structure known as the cavo-tricuspid isthmus.^2^ By directing ablation to interrupt conduction through this region, high cure rates have been achieved with a low risk of complications.^3^

**Figure 1:**
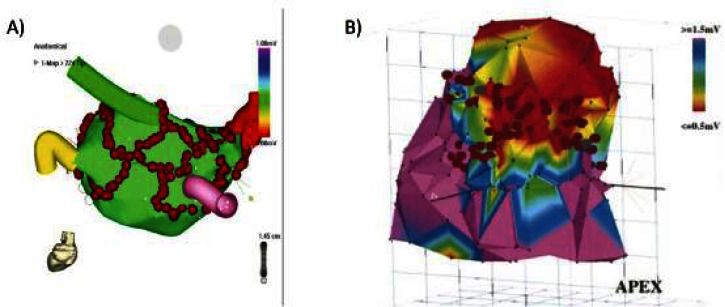
Examples of electrospatial mapping guidance of complex arrhythmia ablation. A and B: Electrospatial surface maps generated by point-by-point contact mapping of the endocardial surface. The red circles are markers where ablation energy was delivered. A: Example of atrial fibrillation ablation in which ablations lesions are placed to encircle the pulmonary veins to prevent exit of arrhythmia triggering foci originating from the pulmonary veins. The pulmonary vein locations are marked by the colored “cartoon” tubes. B: Example of scar based ventricular tachycardia ablation in which linear lesions were placed connecting scar (red) to normal tissue (purple) to interrupt the arrhythmia circuit. Figure 1A included with permission from The Journal of Cardiovascular Electrophysiology. (Calkins JCEP 2005; 13:53) Figure 1B included with permission from Circulation (Marchlinski, Circulation 2000; 101:1288).

**Figure 2:**
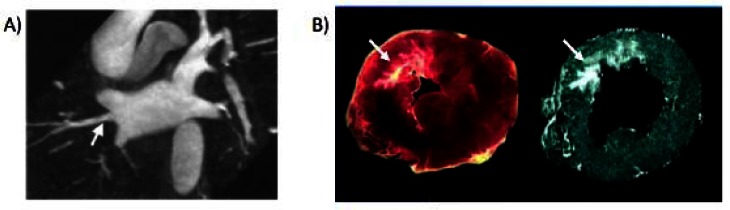
Examples of arrhythmogenic anatomy depicted by MRI. A: MRI angiogram anatomy of the pulmonary veins. Note that variant pulmonary vein anatomy such as an additional right middle pulmonary vein, indicated by the white arrow, can be clearly seen by MRI. B: The complex structure of myocardial infarction scar, indicated by the white arrows, depicted by pathology on the left and delayed gadolinium enhanced MRI on the right. Figure 2A included with permission from the Journal of Cardiovascular Electrophysiology (Mansour, JCEP 2004; 15:387). Figure 2B included with permission from Circulation (Kim, Circulation 1999; 100:1992).

The clinical indications for anatomy-based catheter ablation have since expanded to more complex arrhythmias such as atrial fibrillation and scar-based ventricular tachycardia.^4^,^5^ The basis of these strategies is to target specific anatomic regions and often to create extended ablation “lines” by aligning multiple point lesions or by dragging the catheter along the endocardial surface while applying ablative energy. While the feasibility of X-ray fluoroscopy guidance has been demonstrated for these complex arrhythmias, precise targeting of ablation lesions is limited by fluoroscopy’s inherently poor ability to visualize cardiovascular soft tissue anatomy. Electrospatial mapping systems, which locate the catheter tip in 3-D space relative to magnetic or electric field transmitters, were rapidly adopted to create surface maps of electrical characteristics from multiple regions of the heart and mark the location of ablation attempts so that more elaborate ablation patterns could be created (Figure 1A,B). Electrospatial mapping, however, does not provide direct visualization of the complex underlying arrhythmogenic anatomy (Figure 2A,B). The persistence of sub-optimal cure rates, prolonged procedure and radiation exposure times, and the risk of serious complications have motivated new approaches to facilitate anatomy-based catheter ablation for complex arrhythmias.

Modern imaging techniques such as magnetic resonance imaging (MRI), intracardiac ultrasound, and X-ray computed tomography (CT) are increasingly used to approach the shortcomings of current mapping and ablation systems. Cardiac MRI (CMR) is a particularly flexible imaging modality that offers excellent soft tissue contrast, well characterized gadolinium enhancement techniques for myocardial scar visualization, 3-D imaging of complex cardiovascular anatomy, real-time 2-D imaging along arbitrary imaging planes, and the ability to quantify cardiac motion and blood-flow. This article will review the application of CMR to current clinical procedures and on-going advances toward full CMR guidance of electrophysiology procedures.

## THE PRESENT: ABLATION PLANNING AND GUIDANCE USING PRE-PROCEDURAL CMR

### ATRIAL FIBRILLATION

MRI has been used most extensively to assist planning and guidance of atrial fibrillation (AF) ablation procedures. AF is the most common clinically relevant arrhythmia affecting 0.4% of the general population.^6^ The principal morbidities related to AF are stroke due to embolization of atrial thrombus and symptoms related to poor heart rate regulation with resting heart rates commonly over 110 beats per minute. In the early 1990s surgical modification of the atria with a series of linear incisions was found to be effective at controlling AF, but a minimally invasive catheter-based procedure could not replicate these results.^7^,^8^ It was later recognized that the triggering foci for AF frequently arise from one or more pulmonary veins (PVs).^9^ The ability to cure AF by ablating PV triggers or ablating conduction pathways exiting the PVs was promising but hampered by the risk of pulmonary vein stenosis due to injury of the vessels.^8^ Electrospatial mapping technology led to the development of purely anatomic circumferential ablation strategies in which circular lesions are created further from the PV ostia to block the exit of PV triggers^4^ (Figure 1A). Using this technique alone or in combination with PV isolation, a 70% to 80% success rate has been achieved.^8^ However, repeat procedures are often needed to achieve this success, and the success rate drops to 50% or less for the more chronic forms of AF associated with ischemic, hypertensive, and valvular heart disease.^8^ There also remains a 5% risk of major complications including cardiac perforation, pulmonary vein stenosis, and the rare but potentially lethal risk of atrioesophageal fistula formation.^8^

In an effort to improve procedural success and reduce complications, 3-D MRI angiography (MRA) has been used to assist planning of AF ablation. Kato and colleagues used MRA to study left atrial anatomy in normal subjects and patients with paroxysmal atrial fibrillation and found that 38% of people had pulmonary vein anatomic variants.^10^ Identification of these variants is important because AF-triggering foci can be located within additional veins (Figure 2A). In addition, ablating near small or early branching PVs increases the risk of pulmonary vein stenosis.^11^ Identification of certain anatomic variants before the procedure can also assist in catheter selection or favor using the circumferential ablation approach which is less affected by variant anatomy. Three-dimensional imaging may also reduce the risk for complications by visualizing the relationship of the left atrium to surrounding structures including the esophagus, descending aorta, right pulmonary artery, and left circumflex coronary artery.^12^–^15^ Knowing the location of these structures can be used to direct placement of ablation lesions to lower risk areas or guide reduction of ablation power when lesions are placed close to these structures.

### SCAR-BASED MONOMORPHIC VENTRICULAR TACHYCARDIA

CMR also has the potential to guide the treatment of scar-based monomorphic ventricular tachycardia (MVT), a potentially lethal arrhythmia that is difficult to treat medically or with current ablation techniques. Ventricular tachycardia that results in uniform repetitive electrical activation of the heart arises from anatomically fixed arrhythmia substrate that can be targeted for ablation. Myocardial scarring due to infarction, cardiomyopathy, sarcoidosis, arrhythmogenic right ventricular dysplasia, or cardiac surgery is a common cause of MVT.^16^ Scar-related MVT typically depends on critical isthmuses of conductive tissue bounded by non-excitable scar or a valve annulus.^17^ Ablating isthmus pathways can be curative, but identifying the pathways using traditional mapping techniques can be difficult because these arrhythmias often lead to hemodynamic collapse. Substrate-based approaches utilizing electrospatial mapping to identify reduced voltage scar border zone areas and isolated diastolic potentials within low-voltage scars are now being used to identify critical portions of the arrhythmia circuit to target ablation^5^,^16^ (Figure 1B). Still, ablation of MVT can be arduous. In addition to requiring careful point-by-point electrical mapping of the endocardium, rhythms resulting from epicardial pathways may require additional epicardial mapping, and rhythms resulting from intramural pathways may be inaccessible to electrical mapping. In addition, procedures commonly last over six hours to achieve cure rates in the order of 70% even in the most experienced hands, and success rates can be considerably less in lower-volume centers.

The use of CMR for assisting MVT ablation is still in the investigational stages but shows promise. Delayed enhancement CMR (DECMR) has been used extensively to characterize regions of scar in ischemic and non-ischemic cardiomyopathy (Figure 2B). A number of clinical studies have demonstrated the association of DECMR scar characteristics such as size, transmurality, and border zone area with the risk of MVT.^18^–^20^ Recent work suggests that high-resolution DECMR can be used to assist more directly in MVT ablation planning. Ashikaga and colleagues used an epicardial sock with around 300 electrodes to obtain high-resolution electrical maps during MVT in a pig infarct model.^21^ These maps were registered to very-high-resolution (0.39 × 0.39 × 0.39 mm) *ex-vivo* delayed enhancement MRI (DEMRI) to assess the relationship of MVT electrical propagation to scar morphology. Detailed scar imaging revealed numerous previously unseen features including 3-D tracts of viable myocardium within scar and scar within viable myocardium that visually correlated with the MVT isthmus identified by electrical activation mapping. Ciaccio et al. complemented these findings by computationally predicting suitable locations for MVT ablation with the use of high-resolution DECMR scar imaging.^22^ Using a model that incorporates regional scar thickness to estimate MVT excitation wave-front propagation, the MVT circuit isthmus was predicted and shown to overlap the actual isthmus, observed by electrical mapping, by around 90%. Though these experimental studies use significantly higher-resolution DECMR maps than the typical 1.5 × 2.5 × 8 mm pixel resolution used clinically, methods to obtain higher-resolution scar images in patients are being developed and will be discussed further below.^23^,^24^ Together with studies to identify safe MRI procedures for patients with implanted defibrillators^25^ and clinical correlation of DEMRI scar morphology to successful ventricular tachycardia (VT) ablation sites, the role of MRI for clinical VT ablation should be better defined in the near future.

### THE CURRENT USE AND LIMITATIONS OF PRE-ACQUIRED 3-D IMAGES FOR GUIDING ABLATION

The detailed anatomic information available from CMR is now being used as a road map for guiding placement of ablation lesions.^26^–^30^ A number of techniques have been developed to register electrospatial mapping catheter co-ordinates to pre-acquired 3-D CMR and CT images. Dong and colleagues recently reported their technique in patients undergoing AF ablation.^30^ They felt that 3-D imaging was helpful for tailoring ablations to the variant PV anatomy found in 47% of patients. They also noted that 3-D images of the atria helped to guide lesion placement in areas where stable catheter positioning was difficult, such as along the tissue ridge separating the left atrial appendage from the left pulmonary veins.^11^,^30^ However, even when guided by 3-D image road maps, the study noted that circumferential ablation around the PVs prevented escape of PV triggers in only 32% of patients.^30^ To completely isolate PV triggers, additional electrical mapping and ablation of specific conduction pathways were required. Others have created more extensive, riskier lesions using saline-irrigated ablation catheters and still report PV isolation rates of only 50% to 60% after circumferential ablation around the PVs.^31^,^32^

These sub-optimal results call attention to limitations of this current state-of-the-art in 3-D image-guided cardiac ablation. First, pre-acquired road map images may not correspond to the anatomy during the procedure. Changes in cardiac chamber size associated with variations in heart rate, rhythm, and volume status are not accounted for by pre-acquired imaging and could lead to catheter position registration errors.^29^ Additional registration errors can result from patient motion during the exam, respiratory motion, and beat-to-beat motion of the heart, including significant motion of the PVs.^33^ Second, marking attempted ablation positions and confirming reduction in the local electrogram voltage does not necessarily establish creation of a permanent ablation lesion or a continuous ablation line.^30^ The electrode tissue contact area and electrode exposure to flowing blood are important factors in forming adequate ablation lesions^34^ but are poorly assessed by fluoroscopy and electrospatial mapping-guided procedures. Ablation lesion extent, unintentional gaps in ablation lines, and transient lesion components such as edema similarly are not well predicted by current techniques, including intracardiac ultrasound. These factors limit ablation accuracy and have been shown to reduce procedure efficacy.^35^,^36^

Intracardiac echocardiography (ICE) addresses some of these shortcomings and is increasingly used in clinical practice.^37^ ICE has been used to visualize electrode tissue contact, an important factor for efficient ablation lesion creation. Visualization of microbubbles at the electrode tissue contact interface during ablation has also been used to indicate adequate electrode tissue contact, while the presence of more coarse bubbles has been associated with inappropriately high tissue temperatures that could lead to tissue charring and coagulum formation. However, ICE has limitations for guiding ablation. ICE requires invasive placement and manipulation of a separate imaging catheter, and physical limitations on image plane orientation and field of view limit its ability to evaluate lesion extent and characterize extended regions of ablation. Also the ability of ICE to reliably distinguish regions of ablation from surrounding viable tissue has not been established.

## THE FUTURE: FULLY CMR-GUIDED ABLATION PROCEDURES

There are a number of reasons why intra-procedure CMR is an attractive option for guiding future electrophysiology procedures. First, CMR offers a number of ablation lesion imaging techniques. In addition, the ability to obtain images in arbitrary orientations opens the potential for high-quality visualization of catheters, anatomy, and electrode tissue contact. Further, the position errors introduced by registering catheter position to pre-acquired 3-D images can be largely avoided because both real-time CMR images and 3-D CMR images are acquired in the same co-ordinate system and 3-D images can be reacquired during the procedure if needed.

**Figure 3:**
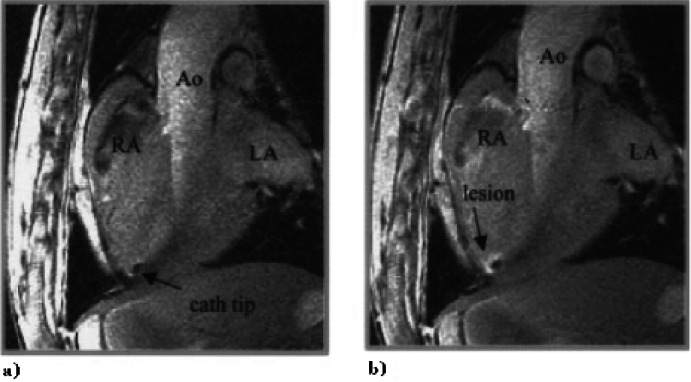
Example of MRI visualization of an ablation catheter positioned at the right ventricle (RV) apex (a) before and (b) after radiofrequency ablation. Post-ablation images were obtained after peripheral injection of gadolinium contrast. Figure included with permission from Circulation (Lardo, Circulation 2000;102(6):698).

Over the last 15 years the basic techniques to enable fully MR-guided electrophysiology (EP) procedures have been developed. Lardo and colleagues introduced the potential of CMR for guiding EP procedures in 2000.^38^ Continuous MR imaging was used to guide non-ferromagnetic EP catheter positioning from an internal jugular vein to selected locations in the right atrium and right ventricle. They also demonstrated the ability to perform and monitor ablations in the MRI scanner (Figure 3A, next page). Delivery of RF ablation energy during imaging can cause significant image degradation, but this noise was dramatically suppressed by 10 MHz cut-off low-pass filtering of the ablation source. After ablation, imaging at the catheter location showed the lesion position and extent using both T2-weighted and gadolinium-enhanced T1-weighted imaging techniques.^38^ The onset of T2 changes at the ablation site was rapid enough that it could be used for lesion monitoring shortly after ablation (Figure 3B).

**Figure 4:**
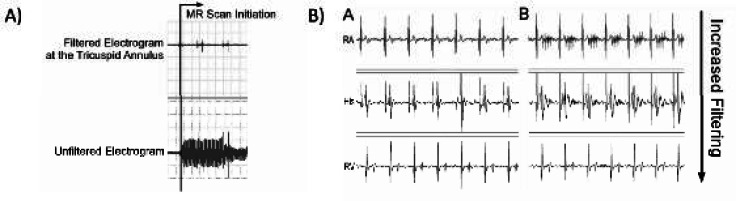
A: Example of the bipolar intracardiac electrograms during scanning before filtering (bottom trace) and after filtering (top trace). B: Example of bipolar intracardiac electrograms at various locations in the heart outside the scanner (left column) and during scanning with increasing levels of filtering (right column). RA = Right atrium, His = His bundle, RV = right ventricular apex.

Subsequent work in our lab has demonstrated the ability to use real-time CMR to perform basic diagnostic EP studies.^39^ Imaging was performed using an unmodified clinical scanner with interactive scan plane manipulation software to guide non-ferromagnetic catheters to standard electrogram recording sites including the high right atrium, His bundle, and right ventricular apex. Electrical interference from gradient switching was adequately suppressed by 30 Hz to 300 Hz band-pass filtering such that even the low-voltage signal from the His bundle could be identified (Figure 4). Importantly, the study demonstrated that MR-guided electrophysiology measurements could be performed safely in human subjects. The topic of device safety in the CMR environment is discussed further below.

Other techniques relevant to EP procedures have also been performed using real-time CMR guidance. Trans-septal catheterization is required for left atrial catheter ablation. While generally safe, the procedure can be difficult in the setting of distorted atrial anatomy and carries the risk of serious complications such as aortic puncture. The ability of real-time CMR to guide trans-septal punctures under direct visualization of the needle, atria, fossa ovalis, and surrounding vasculature has been nicely demonstrated.^40^,^41^ Retrograde catheterization of the left ventricle from the femoral artery is commonly required for VT ablation and has also been performed under real-time CMR guidance.^42^

Recent advances promise to take MR-guided EP from these initial feasibility studies to safe, efficient practice. These advances include 1) intra-procedure ablation lesion monitoring techniques, 2) faster 2-D and 3-D imaging, 3) improved device visualization, 4) intuitive 3-D anatomy and intracardiac electrogram visualization, and 5) MRI-safe device construction techniques.

### INTRA-PROCEDURE LESION MONITORING

Perhaps the most significant advantage of CMR-guided ablation therapy is the potential to visualize ablation lesions with high spatial and temporal resolution. The typical end-point of current ablation procedures is absence of electrical conduction across the ablated region and/or an in-ability to reinduce the clinical arrhythmia with cardiac pacing and medications. However, propagation of electrical signals through the heart is affected by a number of factors including the tissue temperature change induced by ablation.^43^,^44^ Some of these factors may be reversible over time leading to arrhythmia recurrence.^35^,^36^ As described below, CMR appears capable of delineating areas of permanent tissue damage caused by ablation. Using CMR lesion imaging to guide ablation could improve the procedure end-point from assessment of potentially transient electrophysiologic changes to a more direct assessment of complete lines of permanently damaged tissue in the region of interest.

**Figure 5:**
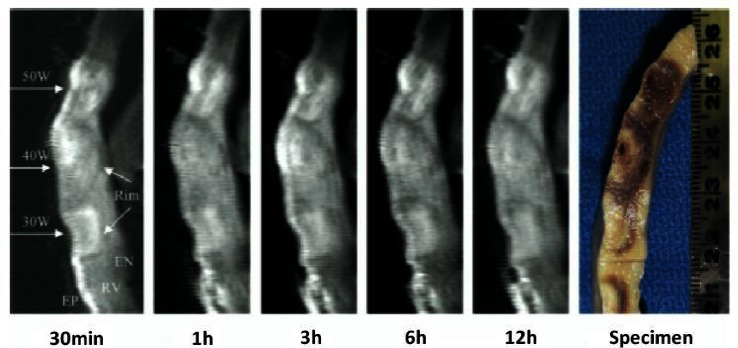
Example of non-contrast T2-weighted MR imaging of right ventricular epicardial RF ablation lesions with pathologic correlation. Stability of the imaged lesion size is demonstrated from 30 minutes to 12 hours after ablation. Figure included with permission from Heart Rhythm (Dickfeld, HR 2007;4(2):215).

A 500 kHz radiofrequency (RF) current is the most commonly used ablation source used for electrophysiology procedures. Cryothermy, ultrasound, laser, and microwave ablation are also being investigated. Ablation lesions can be visualized because CMR is able to detect specific changes in proton precession and relaxation properties resulting from heating and heat-induced biophysical changes in cardiac tissue including interstitial edema, hyperemia, protein conformational changes, cellular shrinkage, and tissue coagulation.^38^ Acute interstitial edema is likely responsible for the hyperintense region corresponding to the area of acute RF ablation damage observed by T2-weighted fast spin echo imaging^38^,^45^ (Figure 5). Dickfield and colleagues found that this hyperintense region correlated well with necrotic lesion size on gross pathology and also noted that gaps between lesions on imaging corresponded with lesion gaps on pathology.^46^ Lesion visualization by T2-weighted imaging has been reported as soon as 2 minutes after ablation, and stable imaging characteristics have been observed from 30 minutes to 12 hours post-ablation.^38^,^46^ This could make T2-weighted MRI a tool to evaluate lesions and lesion continuity over the course of an ablation procedure.

T1-weighted non-contrast-enhanced MR imaging of RF ablation lesions has also been investigated.^46^ T1 changes may be related to tissue heating, protein denaturation, and changes in intracellular and extracellular free water distribution during and following ablation.^45^–^47^ Similar stability of imaging characteristics were reported from 30 minutes to 12 hours following ablation, though the lesion contrast by T1-weighted imaging appears to be less than for T2-weighted imaging.^46^

**Figure 6:**
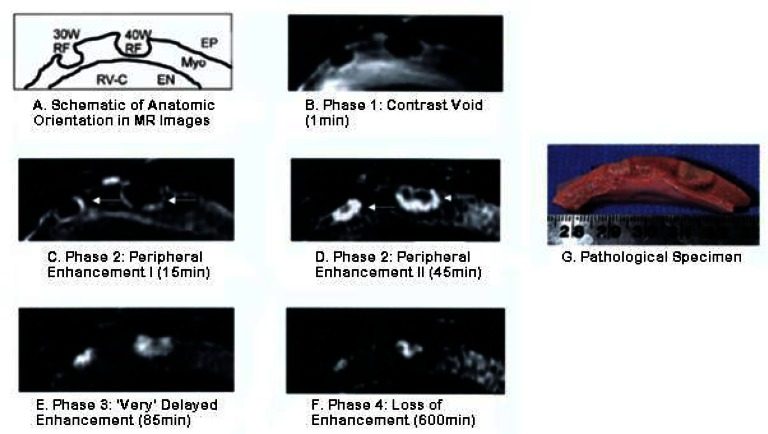
Example of gadolinium-enhanced T1-weighted MR imaging of right ventricular epicardial RF ablation lesions with pathologic correlation. Different lesion enhancement patterns are seen from 1 minute to 2 hours after contrast injection. Figure included with permission from the Journal of the American College of Cardiology (Dickfeld, JACC 2006;47(2):370).

Gadolinium delayed enhancement CMR (DECMR) can provide better visualization of RF ablation lesions compared with non-contrast imaging techniques (Figure 6). The time to achieve full enhancement of RF ablation lesions, 1 to 2 hours, is considerably longer than for DECMR of myocardial infarct scar.^48^ However, good correlation with pathologic lesion size was noted for intermediate enhancement patterns from 1 minute to 2 hours after contrast injection, allowing lesion extent to be assessed without waiting for full enhancement.^48^ The 1 to 2 hour interval required for renal clearance between repeated dosing of gadolinium and the ceiling on total allowable gadolinium dose limit the use of this technique for serial lesion assessment during a procedure.^45^ Still, gadolinium-enhanced imaging may be useful for evaluating gaps in ablation lines after completion of a procedure to assess the need to place additional lesions.

Other methods for monitoring ablation lesion formation during RF energy application are also being investigated. Proton resonance shift thermography is an MRI technique that takes advantage of the decrease in the proton resonance frequency with increasing temperature.^49^ This technique has been used to follow tumor ablation in the uterus, liver, prostate, and brain using diverse energy sources including RF, high-frequency ultrasound, laser, and microwave.^50^–^55^ Its use for following RF ablation in the beating heart is being investigated. Current-vector mapping has also been described for monitoring the extent of tissue power deposition during RF ablation.^56^

While most cardiac ablation lesion MRI studies have been performed in roughly 10 mm thick ventricle, imaging the less than 3 mm thick human atria is of particular clinical interest given the difficulty of achieving long-term pulmonary vein isolation following atrial fibrillation ablation. Peters et al. demonstrated 3-D DECMR of left atrial ablation lesions 10 to 15 minutes after contrast injection using image-based respiratory gating.^24^ This gating technique, also known as respiratory navigator imaging, allowed higher-resolution 3-D imaging to be performed without the need for prolonged breath-holding by tracking diaphragm position on fast 1-D images and collecting 3-D image data within a narrow range of diaphragm positions. Current applications have used a roughly 100 ms mid-diastolic acquisition window timed to precede atrial systole to reduce atrial motion during imaging. Image resolutions of 1.25 × 1.25 × 2.5 mm (reconstructed to 0.6 × 0.6 × 1.25 mm) have been demonstrated. McGann et al. combined this technique with 3-D visualization and quantification to assess left atrial scarring before and following atrial fibrillation ablation. They found that subjects with more than 13% left atrial delayed enhancement following ablation had a nearly 20 times higher chance of being free from atrial fibrillation than those with low amounts of post-ablation delayed enhancement.^57^ The technique is now being used to image immediately following the procedure to target further ablation and potentially reduce the need for repeat procedures. Interestingly, high-resolution 3-D DECMR may also be useful for better identifying patients who will respond poorly to current ablation techniques.^58^ Of note, such high-resolution imaging is not yet feasible in all patients. In the above studies, imaging took 5 to 10 minutes, and 10% to 30% of patients were excluded from analysis because of poor image quality that was attributed to patient motion, significant arrhythmia, or incorrect inversion time selection. Techniques to improve the speed and reliability of high-resolution CMR require further investigation.

### FASTER MR IMAGING

While cardiac gating can be used to generate MR images with excellent spatial resolution by splitting data collection over multiple heart-beats, real-time CMR requires a more deliberate trade-off between temporal and spatial resolution. To visualize catheters adequately, MRI-guided EP procedures require an in-plane spatial resolution of around 2 mm^2^. The target temporal resolution is 7 frames per second (fps), the usual X-ray fluoroscopy frame rate for clinical EP procedures.

Since the initial 1 fps imaging used to guide the first MR-guided EP procedure^38^ faster, stronger gradients have increased the temporal resolution capabilities of fast gradient-recalled echo sequences to the 5 fps range.^59^ Improved gradient performance and B0 field homogeneity have also allowed real-time imaging to be performed with coherent steady state pulse sequences (i.e. steady-state free precession, true fast imaging with steady-state precession (true-FISP), or Fast Imaging Employing Steady-state Acquisition). These sequences provide increased contrast-to-noise performance at a given frame rate compared with fast gradient-recalled echo sequences.^59^ Parallel imaging techniques can provide additional improvement in temporal resolution without sacrificing spatial resolution. These techniques accelerate imaging by covering the region of interest with multiple receive coils and using the different spatial sensitivities of these coils to correct for undersampling of image data.^60^ The acceleration achieved from acquiring less data is countered by the increased processing time required for estimating coil sensitivities and for performing the parallel reconstruction.^61^ Also, for a given number of receive coils, more undersampling leads to deteriorating image signal-to-noise or a reduced ability to suppress aliasing artifacts.^62^ Balancing these issues, 15 fps true-FISP cardiac imaging with 128 phase encode lines can be performed using an 8-channel receive coil array and optimized reconstruction hardware.^63^ Commercial MRI systems now commonly have multichannel receivers and parallel imaging options. The performance of these systems is currently in the range of what is needed to perform CMR-guided EP procedures at 5 fps with acceptable image quality.^61^

While the current imaging rates are adequate for a single 2-D image plane, ideal visualization of the device, target anatomy, and surrounding reference anatomy may require multiple 2-D image planes or even 3-D imaging. Other techniques that can improve imaging speed while balancing imaging quality include non-Cartesian k-space sampling, temporal data sharing between images, and adjusting the trade-off between temporal and spatial resolution.^59^ These techniques may be particularly useful to accelerate imaging of reference anatomy views that are not depended on for device tracking. Use of 32-channel receive arrays to perform more rapid 3-D cardiac imaging and parallel transmission techniques to permit more efficient parallel data collection are also under active investigation.^63^–^65^

### DEVICE VISUALIZATION AND NAVIGATION

While fluoroscopy provides projection images where the entire catheter body and tip are easily visualized, 2-D MR images typically depict a slice through the body that is around 5–10 mm thick. Curved devices such as catheters may pass in and out of the MR imaging plane leading to mis-interpretation of the device tip position. We have noted in preclinical studies that poor delineation of the tip position can result in tissue contact trauma, such as local hemorrhage. In addition, for electrophysiology ablation procedures the device tip contains the energy source. Misestimating the tip/tissue contact region can lead to inaccurate placement of ablation lesions.

During our feasibility studies, tip location has mostly been performed using interactive real-time sequences with a user interface that permits adjustment of the scan plan during image acquisition. Part of the catheter is first identified on some imaging plane, and the plane is manually adjusted until the tip is located. For vascular procedures where the device is constrained to a co-planar segment of blood-vessel, manual plane manipulation is acceptable since only minor image plane translations are needed to visualize the device tip and relevant anatomy. For navigation in cardiac chambers where the device tip location is less constrained, the frequent need for manual plane manipulation necessitates a skilled operator for image plane manipulation and can distract from efficient procedure work flow.

One approach to this problem is to automatically direct imaging to the device location using position sensors located in the catheter. Fifteen years ago Dumolin et al. described using 1-D projection MR imaging along the *x*, *y*, and *z* directions to identify the 3-D position of small receiver coils located in a catheter tip.^66^ This position tracking technique has since been interleaved within real-time imaging sequences to automatically move the image plane position to the catheter tip location during device manipulation.^61^,^67^ While a single tracking coil is sufficient to simply shift the image position, multiple tracking points are needed to maximize visualization of the device body or to orient imaging relative to the catheter tip direction. Multicoil designs and tracking algorithms have been developed to reduce the need for separate matching circuits in space-constrained catheter lumens.^68^,^69^ Other magnetic field and electric field-based position finding techniques have been developed for medical device tracking that could also be used for catheter tracking in the MRI scanner.^70^–^72^ Some of these systems generate both position and orientation information for each sensor assembly.^72^,^73^ These systems may provide more accurate and catheter space-efficient options for device tracking. They can also reduce the performance penalty and avoid the scanner-specific complexities associated with interleaving tracking and real-time imaging sequences.

An alternative technique for device visualization uses non-slice-selective imaging to produce an effect similar to projection X-ray fluoroscopy.^74^,^75^ The non-slice-selective catheter imaging plane can be intersected with slice-selective images containing the target anatomy to assist guidance of the catheter tip. This technique may be used to provide a “fluoroscopy” view of devices using a number of catheter antenna designs^69^,^76^–^78^ (Figure 3A).

Another factor that affects device navigation in the MRI environment is the physical constraint of performing procedures near and within the narrow MRI bore. The availability of shorter, wider-bore, high-field MRI scanners is making this less of an issue. Remote catheter steering is also gaining interest in the EP field to facilitate point-by-point electrical mapping of the cardiac chambers and assist stable device placement during ablation.^79^,^80^ Some of these techniques may be amenable to use in the MRI environment. A robotic catheter manipulation system that uses steerable sheaths with multiple pull wires was recently used to perform atrial fibrillation ablation in patients.^81^ A magnetic remote steering technique has also been described that utilizes the torque generated by current-carrying coils in the static MRI magnetic field to deflect a catheter tip.^82^,^83^

### 3-D ANATOMY AND INTRACARDIAC ELECTROGRAM VISUALIZATION

**Figure 7:**
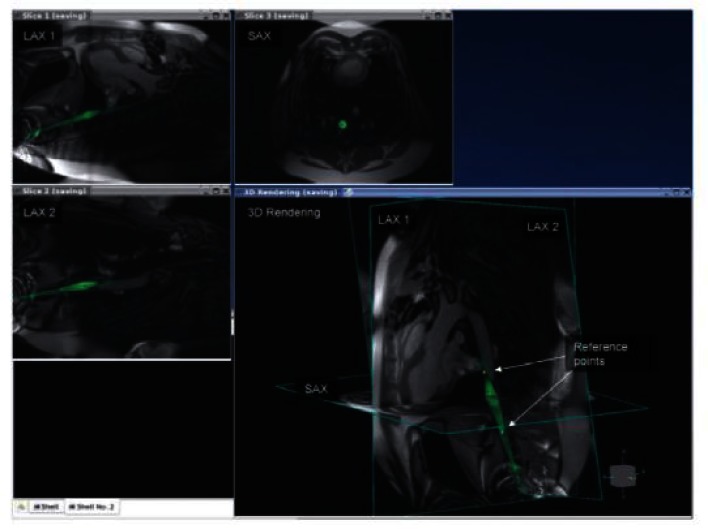
Example of using automatic catheter highlighting and reference image planes to navigate complex 3-D anatomy using real-time MRI. The anatomic location of the catheter position on the image labeled LAX2 is better appreciated when overlaid with long and short axis images of the heart. Figure included with permission from The Journal of Magnetic Resonance Imaging (Guttman, JMRI 2007; 26:1429).

The ability to generate real-time images with arbitrary orientations in addition to anatomically detailed 3-D images with flexible tissue contrast makes CMR well suited for navigating complex arrhythmia anatomy and delineating complex ablation patterns. This flexibility also introduces the potential for disorientation and information overload. Appropriate displays and user-interfaces tailored to the work flow of an EP procedure are needed to manage this flexibility. Because thin-slice real-time imaging can intersect anatomy in unfamiliar ways, 3-D visualizations that plot real-time images oriented relative to reference images can be helpful (Figure 7). A basic imaging interface for MR-guided EP procedures would provide a convenient way to “book-mark” and access reference cardiac views, switch between real-time and lesion visualization sequences during the procedure, appropriately present lesion images for ablation line continuity assessment, and display the relationship of stored images, catheter position, and intracardiac electrogram characteristics in 3-D.

### INTERVENTIONAL MRI DEVICE SAFETY

The most important consideration for any new diagnostic or therapeutic approach is safety. While a number of studies have been performed to determine the safety of conventional MRI with regard to electromagnetic energy exposure and tissue heating,^84^,^85^ interventional procedures add additional considerations and raise new safety concerns.^86^–^89^ The most straightforward aspect to MRI device safety is the avoidance of ferromagnetic materials that could experience significant forces when brought close to the scanner. Though MRI-unsafe objects, such as ferromagnetic scissors and needle drivers, may be needed during the preparatory phase of a procedure, until MRI-compatible alternatives are available a system must be in place to methodically track and remove such objects before approaching the scanner. The electric current associated with defibrillation can also lead to strong displacement forces in high magnetic fields and should be performed with the defibrillator pads maintained a safe distance from the scanner bore.^90^ Similar attention is needed to address the ferromagnetic properties and MRI electromagnetic interference compatibility of other equipment associated with electrophysiology procedures including physiology monitoring equipment, ablation and pacing sources, and anesthesia apparatus. Clear marking of high-field areas and secure placement of objects that may experience magnetic forces is mandatory so that appropriate pieces of equipment are kept at a safe distance.^91^

An additional safety concern particular to CMR-guided cardiovascular procedures is the significant heating that can result from RF transmission-induced current in extended metallic objects such as guide-wires, wired electrodes, and metal-braided catheters.^87^,^88^ This induction is more pronounced when portions of the device are located close to the RF transmit body coil housed within the edge of the scanner bore. Device length is an important factor in efficient coupling and heating; however, many other parameters can influence the sudden onset of significant heating in the setting of an interventional procedure.^88^,^92^ The simplest way to avoid this problem is to construct devices from non-metallic components when possible. Polymer materials for catheter braiding, such as Dacron and Kevlar, and composite materials for guide-wires, such as glass-fiber-reinforced plastics, can be used to achieve device functional characteristics such as torqueability, stiffness, and kink resistance.^38^,^93^ Several approaches have also been developed to avoid significant induction heating in structures that require conductivity. Wires made from high-resistance alloys and gold-sputtered thread have been used to obtain intracardiac electrograms during imaging, with significant reduction of electrode heating.^94^ For structures where efficient power transfer is required, such as pacing or ablation electrodes, high-frequency RF chokes can allow passage of signal lower than a few MHz while blocking unwanted MR transmit frequency currents.^95^,^96^ A promising heating suppression technique for position tracking and intravascular imaging coils that need to pass differential mode signals at the same frequencies as unwanted common mode induced currents is to place thin transmission line transformers in the signal-carrying cables.^77^,^97^,^98^ Other strategies such as detuning and decoupling of circuits prone to heating,^78^,^99^ fiber-optic transmission of signals,^100^ and use of inductively coupled resonators for wireless device tracking^101^ are also considerations for new device design.^25^,^90^ Using surface coils instead of the higher-power body coil for RF transmission has also been proposed as a way to reduce RF current induction in devices.^102^ Now that academic sites and imaging companies are focusing on heating-safe device development, more rapid progress in this area is expected.

Transitioning proof of concept studies to clinical electrophysiology procedures requires collaboration between academic centers, imaging and device companies, and regulatory agencies. The first CMR-guided electrophysiology procedure in a patient was performed using custom catheters made to clinical specifications by a clinical catheter manufacturer.^39^ Prior to use in a human an investigational device exemption was obtained from the Food and Drug Administration (FDA). As part of this device exemption, catheter heating and reduction of heating using RF filtering was demonstrated in a series of device positions and orientations within the scanner using high specific absorption rate (SAR)-imaging protocols.^39^ In addition, safe use of the catheter in animals was documented prior to human studies. Though catheters with embedded imaging coils provided improved device visualization in the animal studies, these devices were not approved for or used in patients. Before generally applying CMR guidance to interventional electrophysiology, more standardized MRI-compatible catheter safety guidelines and device testing protocols need to be developed.

The compatibility of MRI with implanted devices such as pace-makers and implantable cardiac defibrillators (ICDs) is also an important consideration in performing interventional MRI studies in the electrophysiology patient population. Particular concerns include static magnetic field-induced movement of the device and scanning-induced programming changes, device inhibition, activation of tachyarrhythmia therapies, and lead currents leading to heating or cardiac stimulation.^103^–^106^ Modern devices address some of these concerns with the use of less ferromagnetic material and improved resistance to electromagnetic interference.^107^ A number of devices have been carefully studied during *in-vitro* and animal MR imaging, and experience is growing for safe cardiac MRI scanning in patients with selected devices under controlled scanning conditions.^25^,^107^,^108^ This experience includes use of sequences relevant to modern CMR with SAR characteristics similar to those used for real-time MRI.^25^ Still, the number of patients and devices studied thus far is limited, and further work is needed to develop manufacturer protocols for establishing conditional MRI safety of pace-makers and ICDs. Carefully designed protocols for patient selection, monitoring, and scanning also need to be developed before imaging of patients with devices can be more routinely performed. The impact of device-related artifacts on cardiac image interpretation also needs to be more carefully studied.

## CONCLUSION

Increasing knowledge of the anatomic basis for cardiac arrhythmias has extended the role of catheter ablation to curing even complex rhythms such as atrial fibrillation and scar-based ventricular tachycardia. CMR has demonstrated a number of uses for procedural planning, particularly for treatment of atrial fibrillation. The use of DEMRI for planning VT ablation procedures also shows promise. Real-time CMR combined with intra-procedural lesion imaging could allow physicians to accurately guide devices and establish completeness of ablation lines without concern for radiation exposure. This could significantly improve the way current ablation procedures are performed and open the way to ablative cure of arrhythmias such as permanent atrial fibrillation that currently respond poorly to minimally invasive approaches.^7^

## Abbreviations

AF: atrial fibrillation
CMR: cardiac magnetic resonance imaging
CT: computed tomography
DEMRI: delayed enhancement MRI
DECMR: delayed enhancement CMR
EP: electrophysiology
fps: frames per second
ICE: Intracardiac echocardiography
MRA: MRI angiography
MRI: magnetic resonance imaging
MVT: monomorphic ventricular tachycardia
PV: pulmonary vein
RF: radiofrequency
SR: specific absorption rate
true-FISP: true fast imaging with steady-state precession
VT: ventricular tachycardia.
