# Correction for the Article “MRI-Guided Electrophysiology Intervention” by Henry R. Halperin and Aravindan Kolandaivelu

**DOI:** 10.5041/RMMJ.10206

**Published:** 2015-07-30

## CORRECTION

In the following paper: Halperin HR, Kolandaivelu A. MRI-Guided Electrophysiology Intervention. *Rambam Maimonides Med J*, 2010 October;1(2): e00015, it was noted that the Acknowledgement regarding the source of the text was not published. The following Acknowledgement has been added:

**Acknowledgement:** This article is reprinted with slight modifications from Kolandaivelu A, Lardo AC, Halperin HR. Cardiovascular magnetic resonance guided electrophysiology studies. J Cardiovasc Magn Reson. 2009;11(1):21 under the terms of the Creative Commons Attribution License (http://creativecommons.org/licenses/by/2.0).

The HTML and PDF versions of this article have been corrected in the RMMJ.org.il Archives.

